# *PIK3CA* mutations in breast cancer: reconciling findings from preclinical and clinical data

**DOI:** 10.1186/bcr3605

**Published:** 2014-01-23

**Authors:** Dimitrios Zardavas, Wayne A Phillips, Sherene Loi

**Affiliations:** 1 BrEAST Data Center, Insitut Jules Bordet, 1000 Brussels, Belgium; 2 Surgical Oncology Research Laboratory, Peter MacCallum Cancer Centre, Melbourne, Victoria 3002, Australia; 3 Division of Cancer Medicine and Research, Peter MacCallum Cancer Centre, St Andrews Place, East Melbourne, Victoria 3002, Australia; 4 Sir Peter MacCallum Department of Oncology, University of Melbourne, Parkville, Victoria 3002, Australia

## Abstract

*PIK3CA* mutations represent one of the most common genetic aberrations in breast cancer. They have been reported to be present in over one-third of cases, with enrichment in the luminal and in human epidermal growth factor receptor 2-positive subtypes. Substantial preclinical data on the oncogenic properties of these mutations have been reported. However, whilst the preclinical data have clearly shown an association with robust activation of the pathway and resistance to common therapies used in breast cancer, the clinical data reported up to now do not support that the *PIK3CA* mutated genotype is associated with high levels of pathway activation or with a poor prognosis. We speculate that this may be due to the minimal use of transgenic mice models thus far. In this review, we discuss both the preclinical and clinical data associated with *PIK3CA* mutations and their potential implications. Prospective clinical trials stratifying by *PIK3CA* genotype will be necessary to determine if the mutation also predicts for increased sensitivity to agents targeting the phosphoinositide 3-kinase pathway.

## Introduction

Phosphoinositide 3-kinases (PI3Ks) comprise a family of lipid kinases, discovered in the 1980s, that are responsible for mediating important biological functions such as cell survival, differentiation and proliferation [1]. In breast cancer, mutations of the *PIK3CA* gene, which encodes the p110α catalytic subunit of PI3K, are highly frequent (2,257/9,095 = 24.82% according to the Catalogue of somatic mutations in cancer [2]), have been shown to be oncogenic, and are likely to represent important events in the initiation and progression of breast cancer. However, several characteristics of *PIK3CA* mutations in breast cancer have been observed, including a strong association with expression of the estrogen receptor (ER), a lack of an association with robust activation of the classical PI3K pathway, as well as a relatively good prognosis for patients with mutations compared with their wild-type counterparts. These features make it difficult to understand the functional and clinical relevance of *PIK3CA* mutations in breast cancer at present. In this article we review and summarize the preclinical and clinical data in breast cancer in an attempt to reconcile these findings.

## Background

Based on distinct structural characteristics and substrate specificity, PI3Ks can be divided into three classes, I to III. Class I can be further subdivided into class IA and IB kinases, with class IA activated by receptor tyrosine kinases (RTKs), G protein coupled receptors and other oncogenes such as RAS, and class IB activated exclusively by G protein coupled receptors [3]. Class IA PI3Ks represent the most extensively studied subclass, with implications in human carcinogenesis [3]. They are heterodimers consisting of a catalytic (p110) and a regulatory (p85) subunit, with the latter stabilizing the former in quiescent cells and suppressing PI3K activity. There are three different isoforms of the p110 subunit in mammals, p110α, p110β and p110δ, transcribed from the genes *PIK3CA*, *PIK3CB* and *PIK3CD*, respectively, and three isoforms of the p85 subunit, p85α, p55α and p50α, deriving from three genes *PIK3R1*, *PIK3R2* and *PIK3R3*, respectively [4]. The p110α subunit consists of five domains: an amino-terminal domain termed adaptor-binding domain, a Ras-binding domain, a C2 domain, a helical domain and a kinase catalytic domain [5]. The p85α regulatory subunit also contains five domains: an amino-terminal SH3 domain, a Rho-GAP domain and two Src homology 2 (SH2) domains (one towards the amino terminus, nSH2, and one carboxy-terminal, cSH2), separated by an inter-SH2 (iSH2) domain [5].

Upon growth factor stimulation p85 binds through its SH2 domains to phospho-motifs of RTKs, relieving its inhibitory effect over p110 and mediating the recruitment of PI3K to the plasma membrane. The activated p110 subunit catalyses the conversion of phosphatidylinositol-4,5-bisphosphate to phosphatidylinositol-4,5-trisphosphate, which subsequently provides a docking site for the pleckstrin homology domain-containing proteins PDK1 and AKT [6]. The next step is a dual phosphorylation of AKT (on T308 and S473 residues), resulting in its activation and a subsequent intracellular cascade of phosphorylation of other proteins, including mammalian target of rapamycin (mTOR) [7]. The final functional outcome of this cascade of intracellular events is the induction of the multiple biologic effects of the PI3K/AKT/mTOR signaling pathway.

Activation of the PI3K/AKT/mTOR pathway has been demonstrated in all human cancers, with different aberrations variably affecting its different molecular components. In the setting of breast cancer, this represents the most commonly deregulated signaling pathway, with alterations that can be summarized as follows: i) overexpression of PI3K-activating RTKs; ii) inactivating events of negative PI3K pathway regulators (that is, phosphatase and tensin homologue (PTEN) and inositol polyphosphate 4-phosphatase type II); and iii) activating events of PI3K pathway components and/or positive regulators. Mutations of the *PIK3CA* gene, belonging to the third category, represent the most frequently reported molecular alterations of the PI3K signaling pathway in breast cancer.

## Preclinical data

### Oncogenicity of *PIK3CA* mutations

*PIK3CA* has been reported to be mutated frequently in human cancer, particularly in common cancer types such as breast, colorectal, endometrial and prostate [8-16]. This makes it an attractive target for therapeutic intervention. In the setting of breast cancer, *PIK3CA* mutations are extremely common, second only to *TP53* mutations [17-20]. The mutations display a non-random distribution, clustering within the helical domain (exon 9, commonly E542 and E545) and the kinase domain (exon 20, commonly H1047). When first reported, the presence of these ‘hotspot’ positions strongly implied that the mutant protein would be associated with increased kinase activity and oncogenic properties [21]. Such clustering of mutations in specific domains has been noted in other activating oncogenes, such as *BRAF*, *RAS* and *EGFR*. Interestingly, the non-class I PI3Ks have not been reported to be associated with oncogenic mutations.

The function of mutant PIK3CA protein compared with the wild type has been characterized in both human cancer cell lines and human mammary epithelial cells, mainly using gene targeting approaches [22-24]. Several investigators have reported that the mutation was strongly associated with AKT activation, growth factor-independent cell proliferation, resistance to apoptosis, as well as increased invasion and cell migration. Biochemical inhibition of the PI3K pathway was found to be effective in reversing these properties, particularly in *PIK3CA* mutant cell lines [22,23,25,26]. In human mammary epithelial cell lines, the two most common mutant alleles (H1047R and E545K) were found to activate PI3K signaling and could easily form tumors in nude mice [24,26]. Resistance to paclitaxel was also demonstrated [23]. Interestingly, significant increases in tumor angiogenesis have also been reported to be associated with oncogenic *PIK3CA* activity [26].

Differences between the helical and kinase domain mutants have also been extensively investigated. The data suggest that there are at least two different mechanisms by which mutant p110α can activate PI3K signaling. These differences are also supported by structural studies. The helical domain mutants require RAS binding for transformation and are independent of p85, whereas the H1047R mutant depends on p85 binding [27,28]. In another study, helical domain mutants produced a more aggressive phenotype than kinase domain mutants with regard to cellular motility and enhanced extravasation [29]. This study, however, used the MDA-MB-231 breast cancer cell line, which is known to be RAS mutant and ER-negative, so it is conceivable that the helical domain mutant could have synergized with these features. It is unclear how to extrapolate these data when, in breast cancer, *PIK3CA* mutations are strongly associated with an ER-positive phenotype and RAS mutations are extremely rare [29]. As a possible explanation for the phenotypic differences between the various *PIK3CA* mutations, a recent study has reported that helical domain but not kinase domain mutants acquire the capability to interact with IRS1, thus enhancing its ability to associate with the cellular membrane and subsequently activate the pathway [30]. This study highlighted that loss of p85 was not enough to result in growth factor-independent activity of p110α [30] and proposes a mechanistic reason for the differences seen between the helical and kinase domain mutations.

Crystal structure and biochemical analyses have also helped elucidate how different oncogenic *PIK3CA* mutations can change the PI3K architecture and promote oncogenicity dependent on the location of the mutated domain [31,32]. Mutations of the catalytic p110α subunit cluster around the activation loop involved in substrate recognition. In contrast, the helical domain mutants disrupt the interface between p110α and p85α, which likely increases the activity of the enzyme [31,32]. Besides these commonly occurring ‘hotspot’ *PIK3CA* mutations, rarer *PIK3CA* mutations on the C2 and RBD domains have also been found in human cancers. These have mostly been found to also be oncogenic, although due to different mechanisms. For example, mutations in the C2 domain are thought to facilitate p110α localizing to plasma membrane by increasing the positive surface charge of this domain [33].

Interestingly, in breast cancer, the clinical difference between helical and kinase domain mutants is subtle [34,35]. Double mutants, or cases with two different *PIK3CA* mutations, have also been observed in breast cancer, albeit infrequently. There seems to be a higher incidence of *PIK3CA* mutations, particularly the helical domain mutants, in lobular cancer versus ductal invasive breast cancers (lobular 30.8% versus ductal 24.4%; *P* = 0.14) [34]. Also of note is that the common breast cancer cell lines used in preclinical experiments (MCF7 and T47D) contain a *PIK3CA* mutation (helical and kinase domains, respectively). These cell lines strongly express ER, are of the ‘luminal A’ phenotype and are sensitive to treatment with the hormonal agent tamoxifen [36].

### *PIK3CA* mutations and therapy resistance *in vitro*

*PIK3CA* mutations have been reported to be associated with resistance to human epidermal growth factor receptor 2 (HER2) and endocrine therapies in a number of preclinical cell line and xenograft models. In the setting of HER2-positive breast cancer, several preclinical studies have reported that *PIK3CA* mutations are associated with resistance to HER2 blockade with trastuzumab [37,38]. Another study also confirmed that these mutations could mediate resistance to trastuzumab, although the E545K- and H1047R-HER2 overexpressing breast cancer cell lines were sensitive to GDC-0941, a pan-PI3K inhibitor [39]. PI3K signaling pathway activation has also emerged as a molecular mediator of endocrine resistance in the setting of luminal breast cancer, with multiple lines of evidence supporting this notion [40-43]. Several studies have demonstrated a clear synergy between endocrine treatment and various PI3K blocking agents [41-44].

### Mouse models of *PIK3CA* mutations

Generation of transgenic mouse models can help us better understand the function of *PIK3CA* mutation *in vivo*, its contribution to mammary tumorigenesis, as well as its contribution to resistance of commonly used therapies.

Several different types of *Pik3ca*-driven mouse models of breast cancer have been reported (Table 1) [45]. Interestingly, in one study using the MMTV-Cre *Pik3ca*^H1047R^ model high lethality (75%) was observed in mice younger than 4 months due to non-mammary tumor-related causes [46]. Leakiness of the mouse mammary tumor virus (MMTV) promoter resulting in harmful *Pik3ca*^H1047R^ expression in tissues other than mammary gland was thought to be the cause. Similarly, another study with MMTV-Cre *Pik3ca*^H1047R^ mice also showed a high lethality rate for reasons other than mammary tumors (43%), questioning the utility of a broad transgenic method [47]. The other approach has been to create endogenous levels of *Pik3ca*^H1047R^ using a knock-in system under the control of a native promoter (combined with MMTV-Cre) [48,49]. These models are created to induce physiological expression of the mutant protein in the mammary gland only.

**Table 1:** **Genetically engineered mouse models of *PIK3CA *** **mutations**

**Study**	**Mouse model**	**Transgenic versus knock-in**	**Inducible versus non-inducible**	**Penetrance**	**Tumor latency**	**Histology**
Tikoo *et al*. [48]	MMTV-Cre Pik3ca^H1047R^	Site-specific	Non-inducible	100%	Nulliparous mice: 484 days	Fibroadenoma (45%)
						Adenosquamous carcinoma (10%)
					Biparous mice: 393 days	Osteosarcoma (2.5%)
Yuan *et al*. [49]	MMTV-Cre Pik3ca^H1047R^	Site-specific	Non-inducible	NR	Nulliparous mice: 492 days	Fibroadenoma (76.9%)
						Adenocarcinoma (15.4%)
					Multiparous mice: 465 days	Spindle cell neoplasia (7.7%)
Liu *et al*. [50]	MMTV-rtTA TetO-Pik3ca^H1047R^	Transgenic	Inducible (doxycycline)	95%	7 months	Solid (33%)
						Acinar (8%)
						Glandular (5%)
						Papillary (12%)
						Squamous metaplasia (15%)
						Mixed (28%)
Adams *et al*. [47]	MMTV-Cre^NLST^ Pik3ca^H1047R^	Transgenic	Non-inducible	NR	5 months	Adenosquamous carcinoma (51%)
						Adenomyoepithelioma (45%)
						Spindle cell neoplasia (1%)
						Poorly differentiated adenocarcinoma (3%)
	MMTV-Cre^NLST^ Pik3ca^H1047R^; p53^f/+^	Transgenic	Non-inducible	NR	<5 months	Adenosquamous carcinoma (51%)
						Spindle cell/EMT tumor (33%)
						Radial scar lesion (10%)
						Poorly differentiated adenocarcinoma (5%)
Meyer *et al*. [46]	WAPi-Cre Pik3ca^H1047R^	Transgenic	Non-inducible	NR	Nulliparous mice: 219 days	Adenosquamous carcinoma (54.6%)
						Adenomyoepithelioma (22.7%)
					Parous mice: 140.3 days	Adenocarcinoma with squamous metaplasia (13.6%)
						Adenocarcinoma (9.1%)
	MMTV-Cre Pik3ca^H1047R^	Transgenic	Non-inducible	25%	Nulliparous mice: 214 days	Adenomyoepithelioma (100%)

*EMT* epithelial-mesenchymal transition, *MMTV* mouse mammary tumor virus, *NR* not reported.

All the *Pik3ca*-driven models have produced mammary tumors of varying histologies in contrast to single histology mouse models such as Neu, Myc and the polyoma middle-T antigen. These included fibroadenomas, adenocarcinomas, adenosquamous carcinomas, sarcomas and spindle cell tumors. These tumors expressed ERα, as well as basal and luminal cytokeratin markers. Transgenic models resulted in far shorter latency periods, probably due to the overexpression of the mutant and wild-type protein induced by the exogenous promoters. In contrast, the knock-in models, which produce endogenous levels of the mutant protein, had extremely long latencies before the development of tumors, which was shorter in parous versus nulliparous mice, suggesting that pregnancy significantly accelerated *Pik3ca* mutation-mediated mammary oncogenesis. Notably, in one knock-in model, a significant increase in cell number in the ducts (hyperplasia), as well as the number of surrounding stromal cells, was observed [48]. These cells represented expansion of the luminal progenitor population, which demonstrated enhanced colony size and formation, though without signs of classical PI3K pathway activation [48]. The lack of activation of the pathway (pAKT and pS6) seems to more closely replicate the human observations. Overall, metastases have been rarely reported, perhaps suggesting that additional genetic alterations are needed. Two studies reported reduced latencies as a result of synergism between *PIK3CA* H1047R and p53 mutations [47,49]. Another study reported that *PIK3CA* mutant tumors could recur using both PI3K-dependent and -independent mechanisms or c-MET and MYC overexpression, respectively, the latter leading to resistance to a PI3K inhibitor [50].

These data highlight the importance of *Pik3ca* mouse models in contributing to a better understanding of *PIK3CA* mutant pathogenesis and breast cancer development, as well as investigating resistance mechanisms to commonly used therapeutics. They will provide a better understanding of mutation-related cell-extrinsic mechanisms as the tumors grow in the setting of intact immune systems and surrounding stroma. *In vivo* mouse models may perhaps also clarify some of the counterintuitive results that have been observed in the clinical setting, which we will discuss below. Phenotypic differences between knock-in and transgenic models are also evident, and clinical observations will eventually validate which model more closely represents human *PIK3CA* mutated breast cancer.

## Clinical data

### *PIK3CA* mutations, prognosis and treatment efficacy in breast cancer

The clinical relevance of *PIK3CA* mutations in newly diagnosed breast cancer disease has been extensively investigated. Surprisingly, *PIK3CA* mutations have been associated with good prognostic clinico-pathological features in breast cancer. These include positive expression of ER, smaller tumor size and low histological grade [51-54].

Whilst smaller studies initially reported inconsistent prognostic results, the larger studies now emerging seem to be trending in the same direction [40]. The largest published study evaluated *PIK3CA* genotype from 687 tumor samples from patients enrolled in the FinHER prospective, phase III clinical trial [34,55]. *PIK3CA* mutant compared with wild-type patients were noted to have a better prognosis in the first 3 years, which disappeared with longer follow-up [56]. Consistent with these results, a single center retrospective cohort analysis of 590 patients also reported that *PIK3CA* mutations were associated with significantly better clinical outcomes [51]. A retrospective pooled analysis of four neoadjuvant endocrine therapy breast cancer trials involving 278 women did not find that *PIK3CA* mutations were associated with endocrine therapy resistance [57]. Recently, published in abstract form, *PIK3CA* genotyping of the TEAM adjuvant endocrine study found a mutation frequency of 39.8% (1,702/4,272) in post-menopausal patients with ER-positive tumors [35]. Again, significantly better survival was observed for the *PIK3CA* mutant breast cancers: hazard ratio 0.76 (95% confidence interval 0.63 to 0.91), *P* = 0.003.

*PIK3CA* mutations have been reported in ductal carcinoma *in situ*[58], suggesting that they are an early event, consistent with the knock-in mouse models. However, it seems that in breast cancer the mutation is not associated with high levels of PI3K pathway activation such as increased phosphorylated AKT (S473) and pS6 [18,59]. A genomic study reported that a gene signature developed from *PIK3CA* mutant human breast cancers was associated with low mTORC1 output and high *ESR1* signaling [44]. In contrast, *PIK3CA* mutant cell lines were associated with high levels of activation *in vitro*. This observation further supports the use of transgenic knock-in mouse models rather than breast cancer cell lines to investigate the functional effects of *PIK3CA* mutations. These data suggest several possibilities. Perhaps, similar to PTEN deficiency, high levels of PI3K pathway activation could be detrimental to the cell (that is, cause senescence); therefore, strong negative feedback is active in containing pathway activation until a ‘second hit’ disables this [60]. Alternatively, *PIK3CA* mutations may be weak activators of the PI3K pathway, due to the requirement for plasma localization and/or other activating factors, and require another hit(s) for full activation. We also speculate that the mutation may itself activate estrogen signaling given the strong cross-talk that exists between the two pathways. This would result in patients with *PIK3CA* mutations responding well to current endocrine therapies, which may explain the clinical observations.

With regards to HER2-positive disease, a number of single arm, cohort, single institutional series have suggested an association between the PI3K signaling pathway and trastuzumab and/or lapatinib resistance [61-65]. The majority of these have included PTEN loss or *PIK3CA* mutations to define activated PI3K pathway. The only data evaluating differences in treatment benefit from a randomized study did not observe that *PIK3CA* mutations were significantly associated with resistance to trastuzumab [56]. In fact, the opposite was observed. In contrast, in metastatic HER2+ disease, *PIK3CA* mutations have been associated with poor prognosis. Results from a retrospective biomarker analysis in the CLEOPATRA study, a phase III study assessing the trastuzumab, pertuzumab and docetaxel triplet versus trastuzumab, docetaxel and placebo in first-line treatment of HER2-positive metastatic breast cancer [66], were recently presented. *PIK3CA* genotype from the primary (not metastatic) tumor was found to be prognostic, with patients bearing a *PIK3CA* mutation having a worse clinical outcome (*P* = 0.0001) [67]. Interestingly, *PIK3CA* mutations did not predict for resistance to any type of HER2 blockade in this study, with significant clinical benefit of the triple combination of trastuzumab, pertuzumab and docetaxel persisting irrespective of its mutational status [67]. Further data will be required to confirm these findings. A more complete understanding of the genetic composition of these tumors, both primary and metastatic, will also be beneficial. It could be that, in the advanced setting, dual *HER2* amplification and *PIK3CA* mutation results in complete and robust activation of the PI3K pathway.

Hence, it is becoming clearer that *PIK3CA* mutations are associated with better outcomes in primary ER-positive disease. Generating firm associations with prognosis and clinical relevance could perhaps be achieved by a pooled analysis of all available data. This could result in *PIK3CA* genotype being integrated into clinical decision-making. However, its relevance in advanced disease is unclear and may be different from primary disease. However, the most interesting question remains: will a *PIK3CA* mutation predict for increased sensitivity to a PI3K inhibitor?

## Therapeutic targeting of *PIK3CA* mutated breast cancer

**Figure 1:**
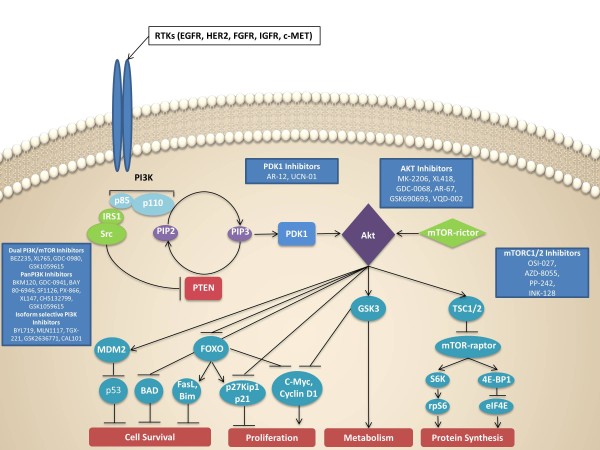
**Schema of phosphoinositide 3-kinase blocking agents currently under clinical development.** Dual phosphoinositide 3-kinase (PI3K)/mammalian target of rapamycin (mTOR) inhibitors (for example, BEZ235, XL765, GDC-0980, GSK1059615), pan-PI3K inhibitors (BKM120, GDC-0941, BAY 80–6946, SF1126, PX-866, XL147, CH5132799, GSK1059615), isoform-selective PI3K inhibitors (p110α selective: BYL719, MLN1117; p110β selective: TGX-221, GSK2636771; p110γ selective: AS-252424; p110δ selective: CAL-101), AKT inhibitors (MK-2206, XL418, GDC-0068, AR-67, GSK690693, VQD-002), mTORC1/2 inhibitors (OSI-027, AZD-8055, PP-242, INK-128), and PDK1 inhibitors (AR-12, UCN-01). PIP_2_, phosphatidylinositol-4,5-bisphosphate; PIP_3_, phosphatidylinositol-4,5-trisphosphate; RTK, receptor tyrosine kinase.

Currently, an abundance of targeted compounds are under clinical development targeting several components of the PI3K signaling pathway (Figure 1, Table 2) [68]. Preclinical evidence demonstrates sensitivity of *PIK3CA* mutated breast cancer cells to PI3K blocking agents [69,70] and, with p110α isoform-selective inhibitors being under clinical development, there is the promise for more potent target inhibition coupled with a milder toxicity profile [71]. Whilst the clinical development of those agents is still too preliminary for any definitive conclusions to be drawn, early data from phase I clinical trials do not support a strong association of anti-tumor activity by pan-class I PI3K blocking agents with *PIK3CA* genotype [72-74]. However, recent early results using the p110α isoform-selective inhibitors look promising in heavily pretreated *PIK3CA* mutant breast cancers [72]. BOLERO-2 was a phase III trial that randomized 724 patients with ER-positive metastatic breast cancer resistant to nonsteroidal aromatase inhibitors to receive exemestane and everolimus (an mTORC1 inhibitor) or placebo. The outstanding results have led to the registration of everolimus in this setting [75]. A biomarker analysis using available primary tumor from 227 (31%) patients from this study and a Foundation Medicine 182 cancer-mutation panel found *PIK3CA* was the most frequently mutated gene among the cases analyzed (48%). However, it was not found to be predictive, with similar treatment benefit derived from the everolimus plus exemestane therapy among *PIK3CA* mutated and wild-type breast cancer patients [76]. Hence, the optimal PI3K pathway inhibition strategy in the setting of *PIK3CA* mutations also remains to be determined.

**Table 2:** **Ongoing clinical trials recruiting breast cancer patients with *PIK3CA *** **mutations**

**Agent**	**Class**	**Trial**	**Description**	**Patients (n)**
BYL719	α-Selective PI3K inhibitor	Phase I (NCT01219699)	Dose escalation in combination with fulvestrant	Postmenopausal women with MBC (160)
		Phase Ib/II (NCT01708161)	Dose escalation in combination with AMG479	Advanced solid tumors (70)
BKM120	Pan-PI3K inhibitor	Phase I/II (NCT01589861)	Dose escalation in combination with lapatinib	HER2-positive, trastuzumab-resistant MBC (106)
MK2206	AKT inhibitor	Phase II (NCT01277757)	Safety and efficacy of MK2206 monotherapy	Advanced breast cancer (40)
		Phase II (NCT01776008)	Safety and efficacy of MK2206 and anastrozole with or without goserelin in the neoadjuvant setting	ER-positive breast cancer, stage II to IIIC (87)
AZD5363	AKT inhibitor	Phase I (NCT01226316)	Dose escalation	Advanced solid tumors and MBC (107)
		Phase I (NCT01625286)	Dose escalation in combination with paclitaxel	ER-positive MBC (110)

*ER* estrogen receptor, *HER2* human epidermal growth factor receptor 2, *MBC* metastatic breast cancer, *PI3K* phosphoinositide 3-kinase.

**Figure 2:**
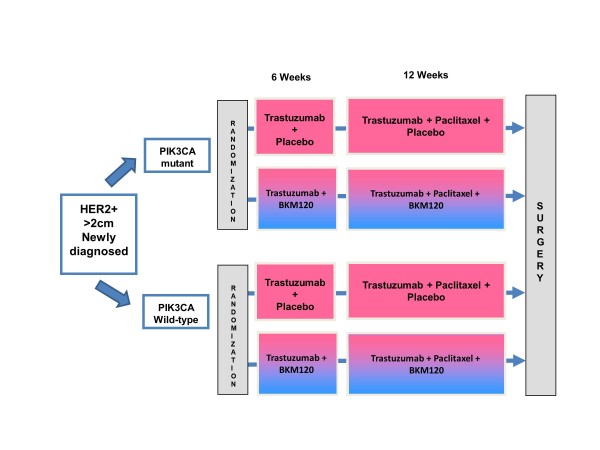
**Trial design of the ‘NeoPHOEBE’ trial with two identical cohorts and upfront *PIK3CA *genotyping to ensure adequate power for both mutant and wild-type cohorts.** HER2, human epidermal growth factor receptor 2.

The only way to definitively determine the prognostic and predictive relevance of *PIK3CA* genotype in breast cancer will be through prospectively defined, upfront stratification in clinical trials. The ‘NeoPHOEBE’ trial (ClinicalTrials.gov study NCT01816594 [77]) is one such trial. This study will evaluate if the addition of BKM120, an oral pan-class I PI3K inhibitor, to trastuzumab improves response rates in HER2-overexpressing breast cancer. Eligible patients will undergo upfront *PIK3CA* genotyping as the trial will essentially have two identical cohorts in order to ensure that the *PIK3CA* mutant population is adequately powered. This trial will attempt to provide answers to the following important questions: i) is *PIK3CA* mutated, HER2-positive disease associated with trastuzumab resistance compared with wild type (prognostic implications), and ii) is *PIK3CA* mutation compared with wild-type associated with an increased response rate in the experimental arm with the PI3K inhibitor (predictive potential) (Figure 2). Only trials such as this one will be able to enlighten us on both the prognostic and predictive implications of this common aberration.

## Conclusion

*PIK3CA* mutations represent one of the most common molecular aberrations in breast cancer. Despite the counterintuitive findings concerning their prognostic significance, active investigation of PI3K pathway blockade is currently ongoing and still could prove to be a curative strategy for *PIK3CA* mutant breast cancers. Prospective clinical trials selecting patients on the basis of *PIK3CA* mutations are currently recruiting (Table 2), but upfront stratification will be required in order to ensure enough power is seen in the *PIK3CA* mutant subgroup. However, there is still much to be learnt about how the mutation contributes to breast cancer growth and, most of all, why high levels of classical PI3K signaling are not observed in human breast cancers. This may be critical to understanding who will respond to therapeutic PI3K inhibition. Recently developed mouse models will help to increase our understanding of cooperating pathways and mammary tumor pathogenesis, as well as immune and stromal influences. Detailed translational research correlative efforts will need to be systematically coupled with clinical trials evaluating efficacy of PI3K inhibitors in breast cancers, as this will enhance our understanding of responders and non-responders by providing the complete genomic landscape associated with *PIK3CA* mutations and treatment response.

## Abbreviations

ER: Estrogen receptor; HER2: Human epidermal growth factor receptor 2; MMTV: Mouse mammary tumor virus; mTOR: Mammalian target of rapamycin; PI3K: Phosphoinositide 3-kinase; PTEN: Phosphatase and tensin homologue; RTK: Receptor tyrosine kinase

## Competing interests

DZ and WAP have no competing interests to declare. A provisional worldwide patent was filed by Université Libre de Bruxelles for *PIK3CA* mutation gene signature: prognosis and therapeutic responsiveness of HER2 overexpressing and estrogen receptor-positive breast cancer. SL is a named inventor, but receives no financial income. SL has acted as consultant for Novartis but has received no honoraria.

## References

[B1] Cantley LC The phosphoinositide 3-kinase pathway Science 2002 296 1655 1657 10.1126/science.296.5573.1655 12040186

[B2] COSMIC: Catalogue of somatic mutations in cancer [http://cancer.sanger.ac.uk/cancergenome/projects/cosmic/]

[B3] Vanhaesebroeck B Stephens L Hawkins P PI3K signalling: the path to discovery and understanding Nat Rev Mol Cell Biol 2012 13 195 203 10.1038/nrm3290 22358332

[B4] Engelman JA Luo J Cantley LC The evolution of phosphatidylinositol 3-kinases as regulators of growth and metabolism Nat Rev Genet 2006 7 606 619 10.1038/nrg1879 16847462

[B5] Amzel LM Huang C-H Mandelker D Lengauer C Gabelli SB Vogelstein B Structural comparisons of class I phosphoinositide 3-kinases Nat Rev Cancer 2008 8 665 669 10.1038/nrc2443 18633356 PMC2847604

[B6] Stephens L Anderson K Stokoe D Erdjument-Bromage H Painter GF Holmes AB Gaffney PR Reese CB McCormick F Tempst P Coadwell J Hawkins PT Protein kinase B kinases that mediate phosphatidylinositol 3,4,5-trisphosphate-dependent activation of protein kinase B Science 1998 279 710 714 10.1126/science.279.5351.710 9445477

[B7] Manning BD Cantley LC AKT/PKB signaling: navigating downstream Cell 2007 129 1261 1274 10.1016/j.cell.2007.06.009 17604717 PMC2756685

[B8] Velho S Oliveira C Ferreira A Ferreira AC Suriano G Schwartz S Jr Duval A Carneiro F Machado JC Hamelin R Seruca R The prevalence of PIK3CA mutations in gastric and colon cancer Eur J Cancer 2005 41 1649 1654 10.1016/j.ejca.2005.04.022 15994075

[B9] Lee J van Hummelen P Go C Palescandolo E Jang J Park HY Kang SY Park JO Kang WK MacConaill L Kim KM High-throughput mutation profiling identifies frequent somatic mutations in advanced gastric adenocarcinoma PLoS One 2012 7 e38892 10.1371/journal.pone.0038892 22723903 PMC3377730

[B10] Spoerke JM O’Brien C Huw L Koeppen H Fridlyand J Brachmann RK Haverty PM Pandita A Mohan S Sampath D Friedman LS Ross L Hampton GM Amler LC Shames DS Lackner MR Phosphoinositide 3-kinase (PI3K) pathway alterations are associated with histologic subtypes and are predictive of sensitivity to PI3K inhibitors in lung cancer preclinical models Clin Cancer Res 2012 18 6771 6783 10.1158/1078-0432.CCR-12-2347 23136191

[B11] Gallia GL Rand V Siu IM Eberhart CG James CD Marie SK Oba-Shinjo SM Carlotti CG Caballero OL Simpson AJ Brock MV Massion PP Carson BS Sr Riggins GJ PIK3CA gene mutations in pediatric and adult glioblastoma multiforme Mol Cancer Res 2006 4 709 714 10.1158/1541-7786.MCR-06-0172 17050665

[B12] Krakstad C Birkeland E Seidel D Kusonmano K Petersen K Mjøs S Hoivik EA Wik E Halle MK Øyan AM Kalland KH Werner HM Trovik J Salvesen H High-throughput mutation profiling of primary and metastatic endometrial cancers identifies KRAS, FGFR2 and PIK3CA to be frequently mutated PLoS One 2012 7 e52795 10.1371/journal.pone.0052795 23300780 PMC3531332

[B13] Riener M-O Bawohl M Clavien P-A Jochum W Rare PIK3CA hotspot mutations in carcinomas of the biliary tract Genes Chromosomes Cancer 2008 47 363 367 10.1002/gcc.20540 18181165

[B14] Qiu W Schönleben F Li X Ho DJ Close LG Manolidis S Bennett BP Su GH PIK3CA mutations in head and neck squamous cell carcinoma Clin Cancer Res 2006 12 1441 1446 10.1158/1078-0432.CCR-05-2173 16533766 PMC1780023

[B15] Kuo KT Mao TL Jones S Veras E Ayhan A Wang TL Glas R Slamon D Velculescu VE Kuman RJ Shih IM Frequent activating mutations of PIK3CA in ovarian clear cell carcinoma Am J Pathol 2009 174 1597 1601 10.2353/ajpath.2009.081000 19349352 PMC2671248

[B16] Kompier LC Lurkin I van der Aa MNM van Rhijn BWG van der Kwast TH Zwarthoff EC FGFR3, HRAS, KRAS, NRAS and PIK3CA mutations in bladder cancer and their potential as biomarkers for surveillance and therapy PLoS One 2010 5 e13821 10.1371/journal.pone.0013821 21072204 PMC2972209

[B17] Banerji S Cibulskis K Rangel-Escareno C Brown KK Carter SL Frederick AM Lawrence MS Sivachenko AY Sougnez C Zou L Cortes ML Fernandez-Lopez JC Peng S Ardlie KG Auclair D Bautista-Piña V Duke F Francis J Jung J Maffuz-Aziz A Onofrio RC Parkin M Pho NH Quintanar-Jurado V Ramos AH Rebollar-Vega R Rodriguez-Cuevas S Romero-Cordoba SL Schumacher SE Stransky N Sequence analysis of mutations and translocations across breast cancer subtypes Nature 2012 486 405 409 10.1038/nature11154 22722202 PMC4148686

[B18] Cancer Genome Atlas Network Comprehensive molecular portraits of human breast tumours Nature 2012 490 61 70 10.1038/nature11412 23000897 PMC3465532

[B19] Stephens PJ Tarpey PS Davies H Van Loo P Greenman C Wedge DC Nik-Zainal S Martin S Varela I Bignell GR Yates LR Papaemmanuil E Beare D Butler A Cheverton A Gamble J Hinton J Jia M Jayakumar A Jones D Latimer C Lau KW McLaren S McBride DJ Menzies A Mudie L Raine K Rad R Chapman MS Teague J The landscape of cancer genes and mutational processes in breast cancer Nature 2012 486 400 404 22722201 10.1038/nature11017 PMC3428862

[B20] Campbell IG Russell SE Choong DY Montgomery KG Ciavarella ML Hooi CS Cristiano BE Pearson RB Phillips WA Mutation of the PIK3CA gene in ovarian and breast cancer Cancer Res 2004 64 7678 7681 10.1158/0008-5472.CAN-04-2933 15520168

[B21] Samuels Y Wang Z Bardelli A Silliman N Ptak J Szabo S Yan H Gazdar A Powell SM Riggins GJ Willson JK Markowitz S Kinzler KW Vogelstein B Velculescu VE High frequency of mutations of the PIK3CA gene in human cancers Science 2004 304 554 10.1126/science.1096502 15016963

[B22] Samuels Y Diaz LA Jr Schmidt-Kittler O Cummins JM Delong L Cheong I Rago C Huso DL Lengauer C Kinzler KW Vogelstein B Velculescu VE Mutant PIK3CA promotes cell growth and invasion of human cancer cells Cancer Cell 2005 7 561 573 10.1016/j.ccr.2005.05.014 15950905

[B23] Isakoff SJ Breast cancer-associated PIK3CA mutations are oncogenic in mammary epithelial cells Cancer Res 2005 65 10992 11000 10.1158/0008-5472.CAN-05-2612 16322248

[B24] Zhao JJ The oncogenic properties of mutant p110 and p110 phosphatidylinositol 3-kinases in human mammary epithelial cells Proc Natl Acad Sci USA 2005 102 18443 18448 10.1073/pnas.0508988102 16339315 PMC1317954

[B25] Kang S Phosphatidylinositol 3-kinase mutations identified in human cancer are oncogenic Proc Natl Acad Sci USA 2005 102 802 807 10.1073/pnas.0408864102 15647370 PMC545580

[B26] Bader AG Cancer-specific mutations in PIK3CA are oncogenic in vivo Proc Natl Acad Sci USA 2006 103 1475 1479 10.1073/pnas.0510857103 16432179 PMC1360603

[B27] Zhao L Vogt PK Helical domain and kinase domain mutations in p110 of phosphatidylinositol 3-kinase induce gain of function by different mechanisms Proc Natl Acad Sci USA 2008 105 2652 2657 10.1073/pnas.0712169105 18268322 PMC2268191

[B28] Chaussade C Cho K Mawson C Rewcastle GW Shepherd PR Functional differences between two classes of oncogenic mutation in the PIK3CA gene Biochem Biophys Res Commun 2009 381 577 581 10.1016/j.bbrc.2009.02.081 19233141

[B29] Pang H Flinn R Patsialou A Wyckoff J Roussos ET Wu H Pozzuto M Goswami S Condeelis JS Bresnick AR Segall JE Backer JM Differential enhancement of breast cancer cell motility and metastasis by helical and kinase domain mutations of class IA phosphoinositide 3-kinase Cancer Res 2009 69 8868 8876 10.1158/0008-5472.CAN-09-1968 19903845 PMC2793177

[B30] Hao Y Wang C Cao B Hirsch BM Song J Markowitz SD Ewing RM Sedwick D Liu L Zheng W Wang Z Gain of interaction with IRS1 by p110α-helical domain mutants is crucial for their oncogenic functions Cancer Cell 2013 23 583 593 10.1016/j.ccr.2013.03.021 23643389 PMC3671608

[B31] Miled N Yan Y Hon WC Perisic O Zvelebil M Inbar Y Schneidman-Duhovny D Wolfson HJ Backer JM Williams RL Mechanism of two classes of cancer mutations in the phosphoinositide 3-kinase catalytic subunit Science 2007 317 239 242 10.1126/science.1135394 17626883

[B32] Huang C-H Mandelker D Schmidt-Kittler O Samuels Y Velculescu VE Kinzler KW Vogelstein B Gabelli SB Amzel LM The structure of a human p110/p85 complex elucidates the effects of oncogenic PI3K mutations Science 2007 318 1744 1748 10.1126/science.1150799 18079394

[B33] Gymnopoulos M Elsliger M-A Vogt PK Rare cancer-specific mutations in PIK3CA show gain of function Proc Natl Acad Sci USA 2007 104 5569 5574 10.1073/pnas.0701005104 17376864 PMC1838453

[B34] Loi S Michiels S Lambrechts D Fumagalli D Claes B Kellokumpu-Lehtinen PL Bono P Kataja V Piccart MJ Joensuu H Sotiriou C Somatic mutation profiling and associations with prognosis and trastuzumab benefit in early breast cancer J Natl Cancer Inst 2013 105 960 967 10.1093/jnci/djt121 23739063 PMC3699437

[B35] Sabine V Crozier C Drake C Piper T van de Velde CJ Hasenburg A Kieback DG Markopoulos C Dirix L Seynaeve C Rea D Bartlett JMS PIK3CA mutations are linked to PgR expression: A Tamoxifen Exemestane Adjuvant Multinational (TEAM) pathology study Cancer Res 2012 72 S1 S5

[B36] Aka JA Adjo Aka J Lin S-X Comparison of functional proteomic analyses of human breast cancer cell lines T47D and MCF7 PLoS One 2012 7 e31532 10.1371/journal.pone.0031532 22384035 PMC3286449

[B37] Berns K Horlings HM Hennessy BT Madiredjo M Hijmans EM Beelen K Linn SC Gonzalez-Angulo AM Stemke-Hale K Hauptmann M Beijersbergen RL Mills GB van de Vijver MJ Bernards R A functional genetic approach identifies the PI3K pathway as a major determinant of trastuzumab resistance in breast cancer Cancer Cell 2007 12 395 402 10.1016/j.ccr.2007.08.030 17936563

[B38] Kataoka Y Mukohara T Shimada H Saijo N Hirai M Minami H Association between gain-of-function mutations in PIK3CA and resistance to HER2-targeted agents in HER2-amplified breast cancer cell lines Ann Oncol 2009 21 255 262 19633047 10.1093/annonc/mdp304

[B39] Junttila TT Akita RW Parsons K Fields C Lewis Phillips GD Friedman LS Sampath D Sliwkowski MX Ligand-independent HER2/HER3/PI3K complex is disrupted by trastuzumab and is effectively inhibited by the PI3K inhibitor GDC-0941 Cancer Cell 2009 15 429 440 10.1016/j.ccr.2009.03.020 19411071

[B40] Zardavas D Fumagalli D Loi S Phosphatidylinositol 3-kinase/AKT/mammalian target of rapamycin pathway inhibition: a breakthrough in the management of luminal (ER+/HER2-) breast cancers? Curr Opin Oncol 2012 24 623 634 10.1097/CCO.0b013e328358a2b5 22960556

[B41] Massarweh S Osborne CK Creighton CJ Qin L Tsimelzon A Huang S Weiss H Rimawi M Schiff R Tamoxifen resistance in breast tumors is driven by growth factor receptor signaling with repression of classic estrogen receptor genomic function Cancer Res 2008 68 826 833 10.1158/0008-5472.CAN-07-2707 18245484

[B42] Juncker-Jensen A Lykkesfeldt AE Worm J Ralfkiaer U Espelund U Jepsen JS Insulin-like growth factor binding protein 2 is a marker for antiestrogen resistant human breast cancer cell lines but is not a major growth regulator Growth Horm IGF Res 2006 16 224 239 10.1016/j.ghir.2006.06.005 16893667

[B43] Miller TW Hennessy BT González-Angulo AM Fox EM Mills GB Chen H Higham C García-Echeverría C Shyr Y Arteaga CL Hyperactivation of phosphatidylinositol-3 kinase promotes escape from hormone dependence in estrogen receptor-positive human breast cancer J Clin Invest 2010 120 2406 2413 10.1172/JCI41680 20530877 PMC2898598

[B44] Loi S Haibe-Kains B Majjaj S Lallemand F Durbecq V Larsimont D Gonzalez-Angulo AM Pusztai L Symmans WF Bardelli A Ellis P Tutt AN Gillett CE Hennessy BT Mills GB Phillips WA Piccart MJ Speed TP McArthur GA Sotiriou C PIK3CA mutations associated with gene signature of low mTORC1 signaling and better outcomes in estrogen receptor-positive breast cancer Proc Natl Acad Sci USA 2010 107 10208 10213 10.1073/pnas.0907011107 20479250 PMC2890442

[B45] Koren S Bentires-Alj M Mouse models of PIK3CA mutations: one mutation initiates heterogeneous mammary tumors FEBS J 2013 280 2758 2765 10.1111/febs.12175 23384338

[B46] Meyer DS Brinkhaus H Muller U Muller M Cardiff RD Bentires-Alj M Luminal expression of PIK3CA mutant H1047R in the mammary gland induces heterogeneous tumors Cancer Res 2011 71 4344 4351 10.1158/0008-5472.CAN-10-3827 21482677

[B47] Adams JR Xu K Liu JC Agamez NM Loch AJ Wong RG Wang W Wright KL Lane TF Zacksenhaus E Egan SE Cooperation between Pik3ca and p53 mutations in mouse mammary tumor formation Cancer Res 2011 71 2706 2717 10.1158/0008-5472.CAN-10-0738 21324922

[B48] Tikoo A Roh V Montgomery KG Ivetac I Waring P Pelzer R Hare L Shackleton M Humbert P Phillips WA Physiological levels of Pik3ca(H1047R) mutation in the mouse mammary gland results in ductal hyperplasia and formation of ERα-positive tumors PLoS One 2012 7 e36924 10.1371/journal.pone.0036924 22666336 PMC3364244

[B49] Yuan W Stawiski E Janakiraman V Chan E Durinck S Edgar KA Kljavin NM Rivers CS Gnad F Roose-Girma M Haverty PM Fedorowicz G Heldens S Soriano RH Zhang Z Wallin JJ Johnson L Merchant M Modrusan Z Stern HM Seshagiri S Conditional activation of Pik3ca(H1047R) in a knock-in mouse model promotes mammary tumorigenesis and emergence of mutations Oncogene 2013 32 318 326 10.1038/onc.2012.53 22370636 PMC3550595

[B50] Liu P Cheng H Santiago S Raeder M Zhang F Isabella A Yang J Semaan DJ Chen C Fox EA Gray NS Monahan J Schlegel R Beroukhim R Mills GB Zhao JJ Oncogenic PIK3CA-driven mammary tumors frequently recur via PI3K pathway-dependent and PI3K pathway-independent mechanisms Nat Med 2011 17 1116 1120 10.1038/nm.2402 21822287 PMC3169724

[B51] Kalinsky K Jacks LM Heguy A Patil S Drobnjak M Bhanot UK Hedvat CV Traina TA Solit D Gerald W Moynahan ME PIK3CA mutation associates with improved outcome in breast cancer Clin Cancer Res 2009 15 5049 5059 10.1158/1078-0432.CCR-09-0632 19671852

[B52] Cizkova M Susini A Vacher S Cizeron-Clairac G Andrieu C Driouch K Fourme E Lidereau R Bièche I PIK3CA mutation impact on survival in breast cancer patients and in ERα, PR and ERBB2-based subgroups Breast Cancer Res 2012 14 R28 10.1186/bcr3113 22330809 PMC3496146

[B53] Pérez-Tenorio G Alkhori L Olsson B Waltersson MA Nordenskjöld B Rutqvist LE Skoog L Stål O PIK3CA mutations and PTEN loss correlate with similar prognostic factors and are not mutually exclusive in breast cancer Clin Cancer Res 2007 13 3577 3584 10.1158/1078-0432.CCR-06-1609 17575221

[B54] Boyault S Drouet Y Navarro C Bachelot T Lasset C Treilleux I Tabone E Puisieux A Wang Q Mutational characterization of individual breast tumors: TP53 and PI3K pathway genes are frequently and distinctively mutated in different subtypes Breast Cancer Res Treat 2012 132 29 39 10.1007/s10549-011-1518-y 21512767

[B55] Joensuu H Kellokumpu-Lehtinen P-L Bono P Alanko T Kataja V Asola R Utriainen T Kokko R Hemminki A Tarkkanen M Turpeenniemi-Hujanen T Jyrkkiö S Flander M Helle L Ingalsuo S Johansson K Jääskeläinen A-S Pajunen M Rauhala M Kaleva-Kerola J Salminen T Leinonen M Elomaa I Isola J Adjuvant docetaxel or vinorelbine with or without trastuzumab for breast cancer N Engl J Med 2006 354 809 820 10.1056/NEJMoa053028 16495393

[B56] Loi S Michiels S Lambrechts D Fumagalli D Claes B Kellokumpu-Lehtinen PL Bono P Kataja V Piccart MJ Joensuu H Sotiriou C Somatic mutation profiling and associations with prognosis and trastuzumab benefit in early breast cancer J Natl Cancer Inst 2013 105 960 967 10.1093/jnci/djt121 23739063 PMC3699437

[B57] Ellis MJ Lin L Crowder R Tao Y Hoog J Snider J Davies S DeSchryver K Evans DB Steinseifer J Bandaru R Liu W Gardner H Semiglazov V Watson M Hunt K Olson J Baselga J Phosphatidyl-inositol-3-kinase alpha catalytic subunit mutation and response to neoadjuvant endocrine therapy for estrogen receptor positive breast cancer Breast Cancer Res Treat 2010 119 379 390 10.1007/s10549-009-0575-y 19844788 PMC2810126

[B58] Miron A Varadi M Carrasco D Li H Luongo L Kim HJ Park SY Cho EY Lewis G Kehoe S Iglehart JD Dillon D Allred DC Macconaill L Gelman R Polyak K PIK3CA mutations in in situ and invasive breast carcinomas Cancer Res 2010 70 5674 5678 10.1158/0008-5472.CAN-08-2660 20551053 PMC2905503

[B59] Stemke-Hale K Gonzalez-Angulo AM Lluch A Neve RM Kuo WL Davies M Carey M Hu Z Guan Y Sahin A Symmans WF Pusztai L Nolden LK Horlings H Berns K Hung MC van de Vijver MJ Valero V Gray JW Bernards R Mills GB Hennessy BT An integrative genomic and proteomic analysis of PIK3CA, PTEN, and AKT mutations in breast cancer Cancer Res 2008 68 6084 6091 10.1158/0008-5472.CAN-07-6854 18676830 PMC2680495

[B60] Carracedo A Alimonti A Pandolfi PP PTEN level in tumor suppression: how much is too little? Cancer Res 2011 71 629 633 10.1158/0008-5472.CAN-10-2488 21266353 PMC3249925

[B61] Razis E Bobos M Kotoula V Eleftheraki AG Kalofonos HP Pavlakis K Papakostas P Aravantinos G Rigakos G Efstratiou I Petraki K Bafaloukos D Kostopoulos I Pectasides D Kalogeras KT Skarlos D Fountzilas G Evaluation of the association of PIK3CA mutations and PTEN loss with efficacy of trastuzumab therapy in metastatic breast cancer Breast Cancer Res Treat 2011 128 447 456 10.1007/s10549-011-1572-5 21594665

[B62] Chandarlapaty S Sakr RA Giri D Patil S Heguy A Morrow M Modi S Norton L Rosen N Hudis C King TA Frequent mutational activation of the PI3K-AKT pathway in trastuzumab-resistant breast cancer Clin Cancer Res 2012 18 6784 6791 10.1158/1078-0432.CCR-12-1785 23092874 PMC3525734

[B63] Esteva FJ Guo H Zhang S Santa-Maria C Stone S Lanchbury JS Sahin AA Hortobagyi GN Yu D PTEN, PIK3CA, p-AKT, and p-p70S6K status: association with trastuzumab response and survival in patients with HER2-positive metastatic breast cancer Am J Pathol 2010 177 1647 1656 10.2353/ajpath.2010.090885 20813970 PMC2947262

[B64] Eichhorn PJ Gili M Scaltriti M Serra V Guzman M Nijkamp W Beijersbergen RL Valero V Seoane J Bernards R Baselga J Phosphatidylinositol 3-kinase hyperactivation results in lapatinib resistance that is reversed by the mTOR/phosphatidylinositol 3-kinase inhibitor NVP-BEZ235 Cancer Res 2008 68 9221 9230 10.1158/0008-5472.CAN-08-1740 19010894 PMC2587064

[B65] Wang L Zhang Q Zhang J Sun S Guo H Jia Z Wang B Shao Z Wang Z Hu X PI3K pathway activation results in low efficacy of both trastuzumab and lapatinib BMC Cancer 2011 11 248 10.1186/1471-2407-11-248 21676217 PMC3141770

[B66] Baselga J Cortés J Kim SB Im SA Hegg R Im YH Roman L Pedrini JL Pienkowski T Knott A Clark E Benyunes MC Ross G Swain SM CLEOPATRA Study Group Pertuzumab plus trastuzumab plus docetaxel for metastatic breast cancer N Engl J Med 2012 366 109 119 10.1056/NEJMoa1113216 22149875 PMC5705202

[B67] Baselga J Cortes J Im S-A Clark E Kiermaier A Ross G Swain SM Biomarker analyses in CLEOPATRA: a phase III, placebo controlled study of pertuzumab in HER2-positive, first-line metastatic breast cancer (MBC) Cancer Res 2012 72 S1 S5 10.1200/JCO.2013.54.5384 25332247

[B68] Sheppard K Kinross KM Solomon B Pearson RB Phillips WA Targeting PI3 kinase/AKT/mTOR signaling in cancer Crit Rev Oncog 2012 17 69 95 10.1615/CritRevOncog.v17.i1.60 22471665

[B69] She QB Chandarlapaty S Ye Q Lobo J Haskell KM Leander KR DeFeo-Jones D Huber HE Rosen N Breast tumor cells with PI3K mutation or HER2 amplification are selectively addicted to Akt signaling PLoS One 2008 3 e3065 10.1371/journal.pone.0003065 18725974 PMC2516933

[B70] Crowder RJ Phommaly C Tao Y Hoog J Luo J Perou CM Parker JS Miller MA Huntsman DG Lin L Snider J Davies SR Olson JA Jr Watson MA Saporita A Weber JD Ellis MJ PIK3CA and PIK3CB inhibition produce synthetic lethality when combined with estrogen deprivation in estrogen receptor-positive breast cancer Cancer Res 2009 69 3955 3962 10.1158/0008-5472.CAN-08-4450 19366795 PMC2811393

[B71] Jamieson S Flanagan JU Kolekar S Buchanan C Kendall JD Lee WJ Rewcastle GW Denny WA Singh R Dickson J Baguley BC Shepherd PR A drug targeting only p110α can block phosphoinositide 3-kinase signalling and tumour growth in certain cell types Biochem J 2011 438 53 62 10.1042/BJ20110502 21668414 PMC3174055

[B72] Juric D Rodon J Gonzalez-Angulo AM Burris HA Bendell J Berlin JD Middleton MR Bootle D Boehm M Schmitt A Rouyrre N Quadt C Baselga J Abstract CT-01: BYL719, a next generation PI3K alpha specific inhibitor: preliminary safety, PK, and efficacy results from the first-in-human study Cancer Res 2012 72 CT-01-CT-01

[B73] Mayer I Abramson V Balko J Isakoff SJ Kuba MG Sanders ME Forero-Torres A Yap JT Van Den Abbeele AD Li Y Arteaga CL Winer E SU2C phase Ib study of pan-PI3K inhibitor BKM120 with letrozole in ER+/HER2- metastatic breast cancer (MBC) J Clin Oncol 2012 30 Abstract 510

[B74] Krop IE Saura C Ahnert J Becerra C Britten C Isakoff SJ Demanse D Hackl W Quadt C Silva AP Burris HA Abu-Khalaf MM Baselga J A phase I/IB dose-escalation study of BEZ235 in combination with trastuzumab in patients with PI3-kinase or PTEN altered HER2+ metastatic breast cancer J Clin Oncol 2012 30 Abstract 508

[B75] Baselga J Campone M Piccart M Burris HA 3rd Rugo HS Sahmoud T Noguchi S Gnant M Pritchard KI Lebrun F Beck JT Ito Y Yardley D Deleu I Perez A Bachelot T Vittori L Xu Z Mukhopadhyay P Lebwohl D Hortobagyi GN Everolimus in postmenopausal hormone-receptor-positive advanced breast cancer N Engl J Med 2012 366 520 529 10.1056/NEJMoa1109653 22149876 PMC5705195

[B76] Hortobagyi GN Piccart MJ Rugo HS Burris HA Campone M Noguchi S Perez A Deleu I Shtivelband M Provencher L Masuda N Dakhil SR Anderson I Chen D Damask A Huang A McDonald R Taran T Sahmoud T Baselga J Correlation of molecular alterations with efficacy of everolimus in hormone receptor-positive, HER2-negative advanced breast cancer: results from BOLERO-2. 2013 ASCO Annual Meeting J Clin Oncol 2013 31 Abstract LBA509

[B77] NeoPHOEBE: Neoadjuvant Trastuzumab + BKM120 in Combination With Weekly Paclitaxel in HER2-positive Primary Breast Cancer [http://clinicaltrials.gov/ct2/show/NCT01816594]

